# Postharvest Disease of Banana Caused by *Fusarium musae*: A Public Health Concern?

**DOI:** 10.1371/journal.ppat.1005940

**Published:** 2016-11-17

**Authors:** David Triest, Marijke Hendrickx

**Affiliations:** BCCM/IHEM Collection of Biomedical Fungi, Service of Mycology and Aerobiology, Scientific Institute of Public Health, Brussels, Belgium; Geisel School of Medicine at Dartmouth, UNITED STATES

## Banana Crown Rot Postharvest Disease

**Fig 1:**
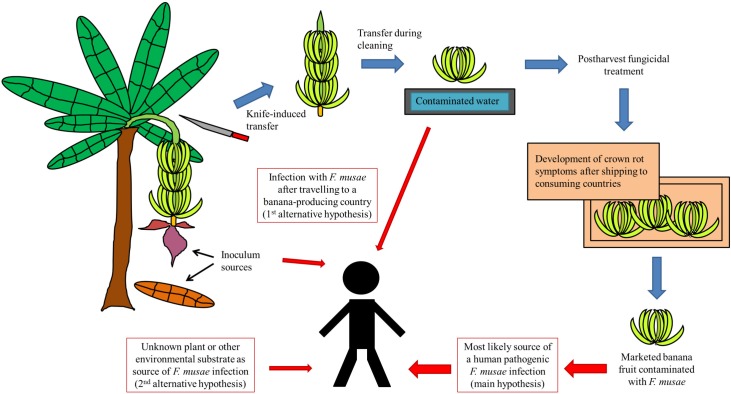
Development of banana crown rot postharvest disease and suggested modes of transfer to cause an opportunistic human pathogenic infection with *F*. *musae*.

Banana is one of the most important tropical crops and is affected by several fungal diseases, such as crown rot postharvest disease [1]. Crown rot is responsible for significant losses in banana fruits [1, 2]. Predominantly, *Colletotrichum musae* and *Fusarium* spp. are its causative agents [1, 2]. Inoculum sources include mainly infected flowers but also decaying leaves, and fungal transfer can occur from banana stalks onto the crown surface during the cutting of banana bunches (knife-induced) as well as when the bunches are cleaned in contaminated water (Fig 1) [1]. Fungal infection starts at harvest, and the first symptoms of crown rot appear only after packaging and shipping from producing countries to consuming countries [1, 2]. Crown rot begins with a mycelium development on the crown surface, followed by an internal development [2]. This internal development can, subsequently, affect the peduncle and the whole fruit, leading to softening and blackening of the fruit tissue [2]. Postharvest fungicidal treatments are applied to control crown rot disease, though severely affected banana fruits are still found in consumer markets [3]. Moreover, onset and spreading of the disease is unpredictable and can also induce early ripening of banana fruits during transport [2].

## *F*. *musae* as Etiological Agent of Banana Crown Rot

Recent studies showed that the fungal species *F*. *musae* is frequently found associated with banana crown rot [1, 3]. The species was installed in 2011 (according to multilocus phylogeny and mating experiments) as a separate, sister species from *F*. *verticillioides* sensu stricto, which is also frequently found associated with banana crown rot [1, 3, 4]. Originally, *F*. *musae* was known as a distinct, banana-infecting population within *F*. *verticillioides*, and both species are practically impossible to distinguish morphologically [4]. Whereas *F*. *verticillioides* has a broad plant host specificity (maize, rice, banana, etc.), *F*. *musae* seems restricted in its plant pathogenicity and has, until now, only been recovered from banana fruits [4, 5]. To date, *F*. *musae* has been isolated from banana fruits coming from several producing countries in Latin America (Mexico, Panama, Ecuador, etc.), the Canary Islands, and the Philippines, but not from banana-producing countries in Africa [1, 4, 5]. Moreover, it has been observed that *F*. *musae* strains have a significantly greater ability to cause infection on banana fruits than *F*. *verticillioides* strains [6]. Another difference with typical *F*. *verticillioides* strains (i.e., isolated from maize) is that *F*. *musae* strains are unable to produce the mycotoxin fumonisin because of the absence of the fumonisin biosynthesis gene cluster [4].

## *F*. *musae* as a Human Pathogen

Because of the recent installment of *F*. *musae* as a separate species (in 2011) and, consequently, the fact that several strains previously identified as *F*. *verticillioides* were actually shown to be *F*. *musae*, its epidemiology is not yet fully elucidated. The latter justifies the need for retrospective studies that reidentify *F*. *verticillioides* strains. One such retrospective study showed that five strains collected in the period 2001–2008 and morphologically identified as *F*. *verticillioides* appeared to be *F*. *musae*, according to multilocus phylogeny [7]. Of interest is that all five strains were clinical isolates, and four were isolated from immune-suppressed patients [7].

In addition, further screening by performing sequence similarity searches in public databases revealed several other cases of human infection associated with *F*. *musae* [7]. These were keratitis cases (i.e., eye infections) from the multistate, contact lens–associated outbreak in the United States as well as superficial infections such as sinusitis [7–9]. Whereas *F*. *verticillioides* is a well-established opportunistic human pathogen, *F*. *musae* was not until this study, which implied its first report as a human pathogen [7]. Another retrospective study reidentifying *F*. *verticillioides* strains showed that human pathogenic *F*. *musae* infections, both deep-invasive and superficial, are probably not as uncommon as previously expected [10], and this is also shown in a one-year survey assessing the identity of *Fusarium* isolates coming from two hospitals in Belgium [11, 12]. It is estimated that approximately 20% of human pathogenic *F*. *verticillioides* infections are in fact infections caused by *F*. *musae* [5]. Important to take into consideration with the latter is that human infections associated with *F*. *verticillioides* account for approximately 10% of the total amount of *Fusarium* infections, and a *Fusarium* infection is considered to be the second most occurring mold infection in humans [13].

## *Fusarium* Virulence Determinants

At least 70 species of the soil-borne fungal genus *Fusarium* are known to be associated with opportunistic human disease, and most of them are members of a species complex [13]. The species complex most frequently found associated with human infection is the *F*. *solani* species complex (between 40% and 60% of the reported cases), followed by the *F*. *oxysporum* species complex and the *F*. *fujikuroi* species complex (each accounting for approximately 20% of the cases) [13]. Both *F*. *musae* and *F*. *verticillioides* are members of the *F*. *fujikuroi* species complex. *Fusarium* spp. are also well-known plant pathogens that are able to infect a diverse range of plant hosts, and several *formae speciales* have been defined. Increasing evidence indicates the presence of host-specific virulence determinants [14]. Despite the availability of whole genome sequences of some important plant pathogenic *Fusarium* spp., knowledge about *Fusarium* virulence determinants remains fragmentary and largely undefined, except for the secreted in xylem (SIX) effectors in *F*. *oxysporum* (i.e., virulence factors secreted by *F*. *oxysporum* in the plant vascular system) and several *Fusarium* mycotoxin encoding regions [14]. Gene expression data from *F*. *oxysporum* f. sp. *cubense* causing banana wilt disease has highlighted an important role for proteins putatively involved in root attachment, cell degradation, detoxification, transport, secondary metabolite biosynthesis, signal transduction, and conidial germination, which is crucial for spreading of the disease in plants as well as for transfer to humans [15, 16]. In addition, comparative genome analyses have revealed that *F*. *solani* f. sp. *pisi* and *F*. *oxysporum* f. sp. *lycopersici* have, in addition to their core genome, an adaptive genome with dispensable chromosomes that are enriched in host-specific genes towards pathogenicity of pea and tomato, respectively. [14, 17, 18]. Also, *F*. *verticillioides* appears to exhibit chromosomes with rapidly evolving genes encoding potential virulence determinants [14]. Moreover, Ma et al. [17] showed that horizontal gene transfer of the pathogenicity chromosomes can convert a non–plant pathogenic *F*. *oxysporum* strain (initially having only a core genome) into a tomato pathogen. Whether *Fusarium* spp. require an adaptive genome to cause a human infection or whether a core genome is already sufficient remains to be elucidated.

## Acquisition of a Human Pathogenic *F*. *musae* Infection

Since *F*. *musae* has only been found on banana fruits, unlike *F*. *verticillioides*, its mode of transfer to cause an opportunistic human infection seems clear and involves an important role for banana crown rot postharvest disease. Although a direct link between a human pathogenic *F*. *musae* infection and a *F*. *musae*-infected banana fruit as the source has not yet been established, two important observations are made: (i) The only known environmental habitat of *F*. *musae* is the banana fruit; (ii) All currently known cases of human infection with *F*. *musae* as the causative agent involve patients hospitalized in non–banana-producing countries (the US and European countries). As such, it is hypothesized that marketed banana fruits contaminated with *F*. *musae* but not yet visibly affected by crown rot postharvest disease most likely lead to an opportunistic human pathogenic *F*. *musae* infection after the susceptible human host is brought into contact with it (Fig 1). However, two alternative hypotheses can be proposed. A first is that the currently known cases of *F*. *musae*-infected patients acquired their infection after travelling to a banana-producing country, where they came into contact with *F*. *musae*-contaminated banana material or cleaning water. The second alternative hypothesis assumes that the habitat and distribution of *F*. *musae* is not as limited as currently described, and *F*. *musae* is also present on currently unknown plant or environmental substrates other than banana.

The *F*. *musae* “banana crown rot” route of infection (i.e., the main hypothesis) deviates from the commonly proposed routes of infection of *Fusarium* species. Because of their efficient mechanisms for dispersal, an opportunistic human pathogenic *Fusarium* infection in the immune-suppressed patient population is often thought to be acquired after inhalation of contaminated (environmental or hospital) air, and their recovery from hospital water–related systems—which act as *Fusarium* reservoirs—also suggests a nosocomial origin [19–21]. Superficial infections in immune-competent individuals, on the other hand, are often acquired after trauma involving tissue breakdowns at the skin and exposure to *Fusarium*-contaminated materials (wood, plant leaves, etc.) [22].

## Future Perspectives

The occurrence of *F*. *musae* as a human pathogen in non–banana-producing countries and its plant host specificity for banana suggests that banana crown rot postharvest disease may be a potential public health concern of importance. The establishment of a direct link between a human infection and a *F*. *musae*-infected banana fruit as the source is one of the major future perspectives. Difficulties for future studies, however, will be the fact that transfer to humans can occur at multiple points (the travel history of the patient will need to be evaluated) and that the potential banana fruit source will often no longer be available for analysis at the time a patient is diagnosed with a *F*. *musae* infection. However, when a potential banana fruit source is still available, *F*. *musae* may be isolated and compared with the clinical isolate by molecular typing methods or whole genome sequencing. Also, it needs to be investigated whether other *Fusarium* spp. associated with banana crown rot postharvest disease, such as *F*. *verticillioides*, are transferred in the same way to cause a human pathogenic infection. Further (retrospective) analyses and clinical surveys, using multilocus sequencing for identification, will need to be performed to fully elucidate the epidemiology of *F*. *musae* infections in banana fruits as well as in humans. Moreover, it needs to be investigated whether *F*. *musae* can be isolated from other plant or environmental substrates, such as hospital water–related systems or air samples. Nevertheless, improvement of postharvest fungicidal treatments to prevent banana crown rot postharvest disease, better control measurements, and better process hygiene criteria for the processing of banana fruits are recommended. In addition, resistant banana cultivars may need to be searched.
